# Explaining integration and differentiation by identifying the rules and coordination mechanisms in a hospital's logistical system

**DOI:** 10.1108/JHOM-06-2020-0236

**Published:** 2021-02-23

**Authors:** Annelies van der Ham, Arno van Raak, Dirk Ruwaard, Frits van Merode

**Affiliations:** CAPHRI, Maastricht University , Maastricht, The Netherlands; Department of Health Services Research, Care and Public Health Research Institute (CAPHRI), Faculty of Health, Medicine and Life Sciences, Maastricht University Medical Centre+ , Maastricht, The Netherlands

**Keywords:** Hospital, Integration, Coordination mechanisms, Rules, Social network

## Abstract

**Purpose:**

Integration, that is, the coordination and alignment of tasks, is widely promoted as a means to improve hospital performance. A previous study examined integration and differentiation, that is, the extent to which tasks are segmented into subsystems, in a hospital's social network. The current study carries this research further, aiming to explain integration and differentiation by studying the rules and coordination mechanisms that agents in a hospital network use.

**Design/methodology/approach:**

The current case study deepens the analysis of the social network in a hospital. All planning tasks and tasks for surgery performance were studied, using a naturalistic inquiry approach and a mixed method.

**Findings:**

Of the 314 rules found, 85% predominantly exist in people's minds, 31% are in documents and 7% are in the information system. In the early planning stages for a surgery procedure, mutual adjustment based on hospital-wide rules is dominant. Closer to the day of surgery, local rules are used and open loops are closed through mutual adjustment, thus achieving integration. On the day of surgery, there is mainly standardization of work and output, based on hospital-wide rules. The authors propose topics for future research, focusing on increasing the hospital's robustness and stability.

**Originality/value:**

This exploratory case study provides an overview of the rules and coordination mechanisms that are used for organizing hospital-wide logistics for surgery patients. The findings are important for future research on how integration and differentiation are effectively achieved in hospitals.

## 1. Introduction

Literature in the field of health care calls for a more integrative approach to the logistical or operational system of hospitals ( Drupsteen *et al.* , 2013 ; Kodner and Spreeuwenberg, 2002 ; Lega *et al.* , 2013 ). There is wide consensus that an integrated perspective in hospitals, which is a central concept in supply chain management, lean strategies and in other operations management theories, can contribute to the improvement of hospital performance ( Aronsson *et al.* , 2011 ; Borges *et al.* , 2019 ; De Vries and Huijsman, 2011 ; Litvak *et al.* , 2008 ; Ludwig *et al.* , 2010 ; Van Merode *et al.* , 2004 ; Villa *et al.* , 2009 ). This approach includes aligning activities and planning resources from the perspective of the total system, taking hospital-wide processes and resources into account ( Aronsson *et al.* , 2011 ). This is considered important in addressing the widely felt need to improve the quality, accessibility and affordability of health-care systems ( Przywara, 2010 ) and of hospitals in particular, given the fact that hospitals are a major cost item in the health-care system ( Morgan and Astolfi, 2015 ).

There are few studies that focus on the impact of adapting integrative practices with regard to improving system-wide performance ( Borges *et al.* , 2019 ). In a previous scoping study ( Van der Ham *et al.* , 2018 ), we found that research on logistics in hospitals typically focuses on one specific logistical flow (patients, material or staff) or on specific departments, but not on the system as a whole. Furthermore, De Vries and Huijsman (2011) point out that little is known on how integration can be achieved in health-care settings.

Ludwig *et al.* (2010) found evidence that more efficient hospitals score high on cooperation, while efficient departments within a hospital do not necessarily contribute to the hospital's overall efficiency. Lawrence and Lorsch (1967) state that not only is integration important, but also that differentiation is essential in order for integration to be effective. They define integration as “achieving unity of effort among the various subsystems in the accomplishment of the organization's task” ( Lawrence and Lorsch, 1967 , p. 4). Differentiation refers to “the state of segmentation of the organizational system into subsystems” ( Lawrence and Lorsch, 1967 , pp. 3–4). Based on these definitions, hospitals that perform well and in which departments cooperate well may have the right degree of integration as well as differentiation in place.

Research in the field of social network analysis (SNA) also addresses integration ( Provan and Sebastian, 1998 ; Kilduff and Tsai, 2003 ; Monge and Constractor, 2001 ). In a previous SNA case study of Slingeland Hospital in the Netherlands, we described the network structure of the logistical system, which includes both integration and differentiation ( Van der Ham *et al.* , 2020 ). This SNA showed that the hospital's network structure differs from its formal organizational structure, with tasks being performed mainly across functional silos and that nurses, physicians and coordinators perform integrative tasks.

However, we agree with Beuving and De Vries (2015) that by reducing human action to structural positions in the network, little is revealed about what actually happens between the agents in the network. We should look specifically at what rules and mechanisms agents use, in order to understand the social network structure, that is, the integration and differentiation observed in the SNA.

Literature pertaining to social networks and integration often refers to coordination between people, groups or organizations as a core activity in organizations ( Provan and Sebastian, 1998 ; Kilduff and Tsai, 2003 ; Monge and Constractor, 2001 ). According to Mintzberg (2012) , there are different types of coordination that connect differentiated activities, which themselves result from the division of labor. Each type of coordination mechanism requires different interactions between agents. Coordination mechanisms are based on rules. Beuving and De Vries (2015) use the metaphor of dancing to explain that dancers follow the rules of dancing that have been set by previous dancers, while at the same time dancers still respond to each other, thereby varying within these rules. Accordingly, the structure of the hospital's social network may be explained by describing the rules that the agents use and by studying the coordination mechanisms that determine the interactions between these agents.

This study deepens the previous SNA study; it aims to explain the integration and differentiation in a hospital by studying the rules and coordination mechanisms that agents use. This topic is important because, despite the fact that there are several promising SNA studies that address the issue of integration ( Provan and Sebastian, 1998 ; Kilduff and Tsai, 2003 ; Monge and Constractor, 2001 ; Uddin and Hossain, 2011 ; Benham-Hutchins and Clancy, 2010 ; Haythornthwaite, 1996 ), Kilduff and Tsai (2003) state that little is known on how coordinating mechanisms facilitate differentiation and integration.

The main research questions are: what are the rules and coordination mechanisms that are used in the hospital's operational system and how do these explain the social network structure, that is, the integration and differentiation?

## 2. Methods

### 2.1 Setting

The study design is based on the case study research method devised by Yin (2014) . Slingeland Hospital was selected for this case study because it is a relatively small Dutch hospital with a highly rated performance and no large transformations took place during the time of research. Additional selection criteria were good access to people and data. Slingeland Hospital has around 1,600 staff members and 120 physicians. It services around 200,000 people in the area and has 350 beds, which is below the average number of 450 beds for Dutch hospitals ( Van Hulst and Blank, 2017 ). Slingeland Hospital performs higher on most logistical indicators than the average Dutch hospital, according to a Dutch benchmark ( Coppa Consultancy, 2012 ). With an average of 89% operating room (OR) utilization in 2016, Slingeland has higher OR utilization than the 82% average of Dutch hospitals that participate in the national benchmark. For other parameters such as lateness and average surgery time, Slingeland performs better than the average hospital that participates in the Dutch benchmark.

### 2.2 Study design

This study was designed to determine what rules and coordination mechanisms are used for the coordination of tasks and why and how this takes place. A naturalistic inquiry approach was followed, aiming to develop a deeper understanding of how the hospital's network functions ( Beuving and De Vries, 2015 ). Data were collected from multiple sources and then analyzed through data triangulation, following a mixed method approach.

**Figure 1:**
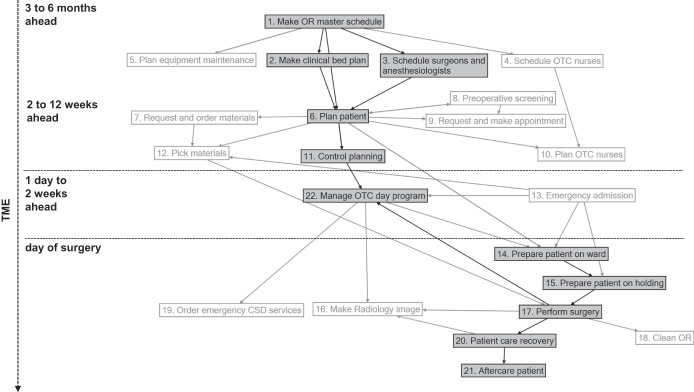
Tasks in scope

The study includes all departments that contribute to either the intake, diagnosis, preparations for or performance of the surgery or the aftercare of surgery patients. Figure 1 shows all the tasks performed, which relate either to the planning or to performing of surgeries, including all activities that take place between patient intake and discharge. In this study, we focus on planning tasks 1, 2, 3, 6 and 11 and tasks directly related to performing surgery, including preparations and aftercare, being 14, 15, 17, 20, 21 and 22. The main reason for this selection is that these tasks involve coordination, as illustrated by the central position of these tasks in Figure 1 . These tasks are performed by agents of outpatient departments, the nursing departments, the Operating Theatre Complex (OTC) and the holding and recovery areas.

The Ethics Committee of Maastricht University reviewed the study design and the data protection aspects of the work that was undertaken. The Committee stated that according to Dutch law, this study did not need a full review of the Ethics Committee, because no humans, that is, patients were involved. Nevertheless, the Committee stated that the work was undertaken in a manner that conformed to the ethics and data protections standards of Maastricht University.

### 2.3 Collected data

Data were collected from four different sources: the Hospital Information System (HIS), documentation, observations and interviews. The collection and analysis of data from the HIS and documentation took place in January 2018 and March 2019. Observations and interviews took place between March 2018 and June 2019.

HIS data were collected in order to determine the system's output, to observe the system's rhythm and what resources were used for surgery patients. The HIS data include registrations of surgeries performed in 2018, including date of surgery, resources involved and timestamps of different stages in the surgery patient's process and in which nursing wards patients stayed before and after surgery.

Documentation was collected in order to find rules that are written down. In total 55 documents were collected, including management reports, planning schemes, working procedures, emails and internal presentations. In addition, planning rules were listed between February and May 2019 by one outpatient secretary and the clinical bed planner. This activity was part of the preparations for the implementation of a central planning department in which surgeries are planned, as of June 2019, by central planners.

Planning and controlling activities were observed over 18 observation days in order to find additional local documents on working procedures and any unwritten rules. The 18 observations took place at three outpatient departments, three nursing departments, the holding area, three surgeries in the OR, the recovery area, with the OTC day coordinator, at the preoperative screening department, two planning meetings and twice at the workplace of the clinical bed planner. During each observation, the activities of the hospital staff were observed and several unplanned informal conversations with staff took place, as they explained what tasks they performed. The sequence of events for each observation, together with relevant parts of the conversations, were reported in an observation report.

All collected data were then further explored in 25 interviews, looking specifically for unwritten rules. For the interviews, we selected people who are involved in planning activities, including the application controller of the OR, the OTC capacity planner, 11 secretaries of the various outpatient departments, the OTC day coordinator and one cluster manager. For each interview, a topic list was prepared, including questions on rules, rhythm, interaction and performance. In addition, a data dashboard was prepared for the interviews with the outpatient secretaries. The dashboard includes HIS data on the number of surgeries and surgeons, planned and emergency surgery percentages, the yearly and weekly pattern of sessions and surgeries, the waiting list development, the percentage of types of surgeries that are performed by one or multiple surgeons and variations in surgery time. In addition, a table was prepared including the average age of patients, utilization rates of OR sessions, the deviation percentage between planned surgery time and actual surgery time, the number of surgeries with particular planning rules, how many patients stayed on each nursing ward and which anesthesiology techniques were used. These data were used in the interviews in order to find unwritten rules and deeper explanations for how agents act. All interviews were recorded and transcribed ad verbatim.

### 2.4 Data analysis

All 94 qualitative data sources, that is, documents, observations and interviews were structured in five data matrices that include the three main topics of rules, rhythm and interaction. One data matrix was constructed for each of tasks 1, 2 and 3. One data matrix was made for tasks 6 and 11 and another for tasks 14, 15, 17, 20, 21 and 22, because with regard to coordination activities, these tasks are strongly connected.

The rules that are used for each task, that is, from each data matrix, were then listed. First of all, for each rule the sources in which the rule was found were registered. Rules were then labeled as hospital-wide or local. A hospital-wide rule is used throughout the entire hospital system and a local rule exists for one particular department, group of people or person. In order to assess whether rules are written or unwritten, we indicated whether the rule itself is registered (R) or the output of applying the rule is registered (O) in a document or in the HIS, or if it exists in the mind of hospital staff.

Furthermore, one or multiple coordination mechanisms through which each rule in the hospital is applied were registered. Coordination mechanisms include (1) mutual adjustment, (2) direct supervision and standardization of (3) work processes, (4) output, (5) skills and (6) norms ( Mintzberg, 2012 ). A rule is applied through “mutual adjustment” if an agent interacts with other agents regarding what a rule entails or if the rule is applied through communication in a specific situation. There is “direct supervision” if a rule is set and monitored by people with formal authority. Rules are the result of “standardization of work” when they result from specified or programmed working processes. They are related to “standardization of output” when rules include specified output in terms of predetermined standards for services or performance. When coordination results from rules regarding specified skills and knowledge, this is labeled as “standardization of skills.” Finally, when rules result from a common culture or ideology, they relate to “standardization of norms”, in which case rules related to behavior are set.

The classification of the rules and coordination mechanisms was validated by a second researcher. He reviewed all rules and the associated coordination mechanisms, based on Mintzberg (1979) and an agreed set of criteria for operationalization of those mechanisms. Rules that were classified differently by the reviewer were then discussed by the prime researcher and the reviewer. During the discussion, the differences in interpretations of Mintzberg's definitions of coordination mechanisms were corrected or the classification was substantiated with the collected data. Consensus was reached on all rules with regard to the coordination mechanism that is used for a rule.

## 3. Results

### 3.1 Output of the network

**Figure 2:**
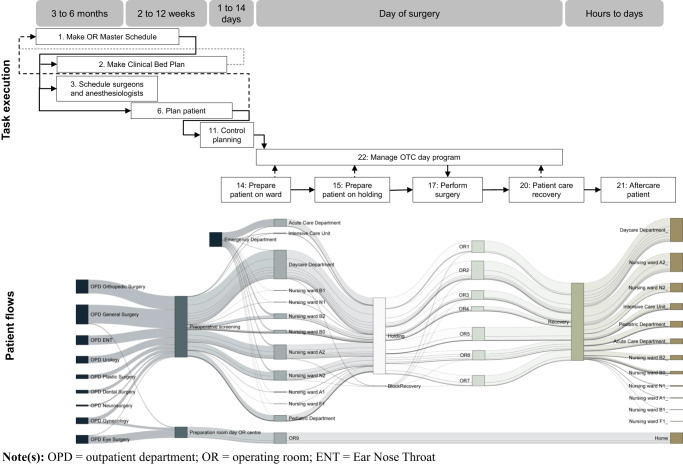
Performed tasks and patient flows over time

In 2018, a total of 9,846 patients who required surgery were diagnosed in one of nine outpatient departments. In total 344 different types of surgery procedures were performed, by 48 different surgeons and 14 assistant surgeons in eight different operating rooms. Patients were cared for in 12 different nursing departments. Of all surgeries, 82% were planned beforehand, that is, they were not emergency surgeries. Patients flowed through a series of locations, as shown in Figure 2 .

There is a variable rhythm in the system, as shown by Figures A1 and A2 in Appendix A . In 2018, the number of surgeries varied from a minimum of 20 to a maximum of 242 surgeries a week, as shown in Figure A1 in Appendix A . The number of surgeries per week varies by 25% on average. Per medical discipline, variability is larger with an average relative standard deviation of 41% (parameter 12 in Table 3 . Types of surgery procedures were performed between 1 and 729 times per year in 2018, with an average of 23 times. Of all types of surgeries, 5% were performed once a week or more on average.

### 3.2 Tasks and flows

The main task of the logistical system is to get the right patient, surgeon, anesthesiologist, nurses, materials and infrastructure together at the right time and in the right place. In order to succeed in this, planning and scheduling of resources in relation to patient demand take place, as presented by tasks 1, 2, 3, 6 and 11. Everything that has been planned is performed on the day of surgery through tasks 14, 15, 17, 20, 21 and 22. All tasks are described in more detail in Appendix B .

In the OR master schedule (task 1) and clinical bed plan (task 2) operating time, space and beds are allocated to medical disciplines. After the OR master schedule is set, surgeons and anesthesiologists schedule when they will work in the outpatient department and in the OR (task 3). The patient is planned for in task 6, along with the surgeon who will perform the surgery. In task 11, the final OTC planning is checked and revised. After that, the patient enters the hospital for the surgery, and a series of tasks are performed from the intake of the patient (task 14) until aftercare (task 21).

Tasks are differentiated on the basis of multiple rules. First of all, tasks throughout the patient process are allocated to different departments. In outpatient departments the patient is diagnosed and planned for. The intake, preparations and aftercare take place in nursing wards, and actual surgeries are performed in the OTC. Second, the care for patients is differentiated according to medical disciplines. This is most visible at the start of the process, when the patient enters the hospital in one of the outpatient departments, each of which is associated with a medical discipline. For nursing wards, the medical discipline, the expected the length of stay (e.g. the Daycare Department), the age (e.g. the Pediatric Department) and the acuity (e.g. the Intensive Care Unit) of the patients are differentiation criteria.

As a result of differentiation, in 2018, patients flowed past the locations shown in Figure 2 , using 122 different routes. Each route is a unique combination of either an outpatient department or the Emergency Department to a ward, the OTC and a ward for aftercare. Table A1 in Appendix A shows how many patients from each medical discipline flowed past each location.

Figure 2 shows that planning tasks 1, 2, 3, 6 and 11 are performed in parallel. The OR master schedule provides input to the planning for beds (task 2), to the schedule for surgeons and anesthesiologists (task 3) and to the planning of patients (task 6). Patients enter the hospital for an unknown number of weeks before the surgery takes place and are registered in the surgery planning between 2 and 12 weeks ahead of the surgery day, with an average of 45 days. So, the OR master schedule, the clinical bed plan and surgeon's schedules are set before the patient demand is known. In addition, because surgeon's schedules are not shared with agents outside the medical disciplines, information on when surgeons operate emerges from task 6. As a result, the OR master schedule and clinical bed plan are adapted and there is feedback between tasks 6, 2 and task 1, as illustrated by the dotted lines in Figure 2 . On the day of surgery, tasks are performed in a fixed order, which is based on how patients flow from one location to the other, as shown in Figure 2 . Controlling this chain of events is done largely in the OTC (task 22) by the OTC day coordinator, who receives feedback from tasks 14, 5, 17 and 20.

### 3.3 Rules and coordination mechanisms

In Appendix B , the rules, coordination mechanisms and interactions that are used for performing the tasks are listed. Table 1 shows that 31% of the total 314 rules are captured in documents; 7% are in the HIS, but most rules (85%) predominantly exist in the minds of the agents in the network. Rules can both be written down and reside in people's minds, in which case they are written down in an often local document and shared throughout the hospital through social interaction. In total 82% of the documents are not generally known, as these are local or personal documents such as checklists, emails, memos or delivered in internal presentations. Besides being in documents, 16% of all rules are more or less written down because the consequence of applying a rule is registered in the HIS, as, for example, the OR master schedule.

Tasks are mainly coordinated through standardization of work (67%) and mutual adjustment (49%) as shown by Table 1 . Other rules refer to output (18%), and a minority of rules relate to skills (4%) or norms (2%). There is no direct supervision. For 38% of rules, more than one coordination mechanism is used, and this explains why the total percentages do not add up to 100%. In particular, on the day of surgery (tasks 14–22), standardization of both working procedures and output go hand in hand with mutual adjustment. For example, rule 285 states that the nurse anesthetist takes the patient to the recovery area and executes the Time Out Procedure (TOP) with a recovery nurse. The TOP is coordinated by standardization of work and output, because both the process and required output are specified. The TOP has to be performed through mutual adjustment between the nurse anesthetist and the recovery nurse, because they perform the TOP together, thereby responding to each other.

In the early stages of planning (tasks 1 and 2), mutual adjustment using hospital-wide rules is the dominant coordination mechanism, whereas closer to the day of surgery there is more standardization of work, based on both hospital-wide and local rules. On the day of surgery, there is also standardization of output, which includes hospital-wide rules that need to be met in order for the patient to be operated on.

### 3.4 Coordination and interaction per task

Tasks 1 and 2 are performed using mainly hospital-wide rules in a highly connected social network structure in which almost all agents interact with one another, as shown by the interaction matrices in Appendix B . Hospital-wide rules are largely based on space and time structures that are largely taken for granted by all agents in the hospital. The time structures relate to universal and national time structures, such as the distinction between weekdays and weekends, public holidays and annual national conferences for physicians. In addition, time is structured by defining working hours and by allocating operating time on the basis of historically acquired rights to operating time by medical disciplines. Space structures result from the infrastructure, that is, the physical building and the presence or absence of specific equipment in operating rooms. As a result, the OR master schedule remained largely unchanged throughout 2017 and 2018; 82% of the allocated OR sessions in the first quarter of 2017 are identical to those in the last quarter of 2018.

In addition, hospital-wide rules define how to negotiate through mutual adjustment on allocated operating time, which is defined in OR sessions and on beds. Both of these have to be requested or returned via the OTC capacity planner and the clinical bed planner. There are norms and output measures as to how and how often a medical discipline may or may not request or return OR sessions or beds, but these are discussed or put aside under certain circumstances, through mutual adjustment. Table 3 shows that most medical disciplines request and return OR sessions. As a result, in 50 weeks in 2018, the OR master schedule was different from the initial schedule. The initial OR master schedule and clinical bed plan, which are recorded in Excel and printed on paper, deviate from the allocated OR sessions and beds that are registered in the HIS.

Surgeons and anesthesiologists are scheduled within each medical discipline (task 3), thereby predominantly following local rules. These rules are not set in the HIS, but in local documents, computer systems or through oral agreements between agents. Documents or systems are not shared hospital-wide nor are they easily accessible to agents outside the outpatient department. There is a multitude of locally defined scheduling methods, for example, surgeons are either scheduled on fixed days or not, perform surgery part of the day or all day, distribute shifts in different ways and so on. These rules are set and applied within each medical discipline through mutual adjustment.

When surgeries for patients are actually scheduled (task 6), the overview of the inflow of patients and what resources are needed starts to build up. Hospital-wide rules state that medical disciplines plan patients using their own methods (e.g. rules 158 and 165), using standard surgery coding and surgery times set in the HIS. Within the outpatient departments, the secretaries match the patient's wishes and the surgeon's orders, thereby following a multitude of local rules with regard to demand management, resource allocation to surgeries and national regulations and norms. For 80% of the 344 types of surgery procedures, one or more specific rules are used; this includes 52% of all performed surgeries. Rules relate to individual patients as well as individual surgeons, as illustrated by rules 147–157 in Appendix B . Secretaries often use a preliminary planning schedule, which is mostly kept in personal paper notebooks, on whiteboards or in the secretaries' minds, before registering the surgery in the HIS. By looking at the HIS and by interacting with the outpatient secretaries and surgeons, the OTC capacity planner and the clinical bed planner monitor the planned patients and try to control the overall planning in order to prevent any possible instability, for example, shortages of beds, cancellations of surgeries or lateness in the OTC. In the two weeks before the surgery date, the preoperative secretaries, the outpatient secretaries and the OTC capacity planner and ward team leaders meet up to check whether all rules have been met in order to proceed with the surgeries as planned (task 11).

Tasks that are performed on the day of surgery include mainly hospital-wide rules that result from the TOP, which is a series of standard checklists that is used in every step of the patient process, from intake until discharge, and which is laid down in the HIS. What needs to be done for patient transfer is standardized and registered in the HIS, but exactly when and by whom activities are performed is determined during task performance, through mutual adjustment. In order to maintain stability, the OTC day coordinator monitors the progress of surgeries, both by looking at the HIS and by observing the holding area, the recovery area and ORs. He uses several local rules for detecting any potential factors that may endanger a smooth patient flow in the OTC.

### 3.5 Rules, coordination and interaction over time

In the planning process, initially drafted hospital-wide schedules are adjusted as time passes and reality unfolds. Table 2 shows that 3–6 months ahead, coordination is mainly based on hospital-wide rules (75%), through standardization of work (67%) and mutual adjustment (66%). Then, from three months to one week before surgery, 40% of the rules applied are local rules and 68% are part of standard working procedures. In this phase, the OTC capacity planner and the clinical bed planner coordinate local planning through mutual adjustment. On the day itself, there is predominantly hospital-wide standardization of work and output (see Table 2 ).

**Figure 3:**
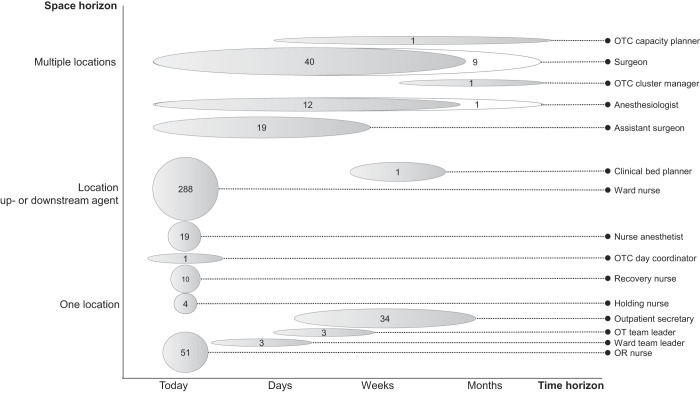
Time and space horizon of the agents

Figure 3 shows that more agents become involved in the interaction as the surgery date approaches. Twelve agents are involved from the start (tasks 1 and 2), six months ahead, and 373 agents interact only on, or about, the day of surgery. Besides a time horizon of one day, these 373 agents have a space horizon of one department or they go one step up- or downstream, for example, a ward nurse who takes the patient to the holding area. Surgeons and one anesthesiologist are involved from months before until the day of surgery, and they work in multiple locations in the hospital. As shown in Figure 3 , over the long term (task 1), nine surgeons and one anesthesiologist are involved in coordination. In the short term, they coordinate on the day of surgery (tasks 14, 15, 17, 20, 21, 22). In between those two time horizons (tasks 3 and 6), surgeons and anesthesiologists coordinate locally.

## 4. Discussion

In a previous SNA study ( Van der Ham *et al.* , 2020 ), we described the Slingeland Hospital's social network structure, that is, the integration and differentiation that shape the network. In the current study, the social network structure of Slingeland Hospital was further explored by studying the rules and coordination mechanisms that explain the interactions observed in the social network analysis.

In order to coordinate the patients' process from hospital admission to discharge, and to schedule the use of all necessary resources, 314 rules were found. Long-term schedules and plans are the result of applying hospital-wide rules, which were set in the past and by time and space structures. In the shorter term, these schedules are subsequently adapted to the circumstances through negotiation by agents in the social network. Circumstances unfold when the local schedules for surgeons are made and when patients are planned for surgery in the OTC. Standardized ways of working are adapted to the circumstances as they present themselves, thus requiring mutual adjustment. There is continuous interaction between agents to observe the expected future and current state of the system, which often changes given the variable rhythm of the system. In addition, there is no reliable central view of all local rules and schedules, as these reside mostly in people's minds. Most agents interact shortly before or on the day of surgery.

The OR master schedule, the clinical bed plan and the surgeon's schedules create open loops, because they are not based on future patient demand. As time passes, the loops are closed through mutual adjustment, by adapting these schedules to the reality of the actual demand and availability of resources. The OTC capacity planner and the clinical bed planner continuously monitor the current situation and raise issues that may endanger the stability of the system with other agents, that is, outpatient secretaries and surgeons. Any remaining open loops on the day of surgery are closed by the OTC day coordinator, nurses, nurse anesthetists, surgeons and anesthesiologists.

Central to literature pertaining to surgery planning is the idea of being able to structure time, space, demand, resource availability and uncertainty ( Demeulemeester *et al.* , 2013 ; Hulshof *et al.* , 2012 ; Zhu *et al.* , 2019 ). Many studies use formal and mathematical methods to create a controllable future state of the system. In contrast, in this case agents work in a negotiated order ( Parhankangas, 2005 ), in particular for planning tasks. Copp *et al.* (2005 , p. 525) state that in negotiated orders, there are “ongoing processes of negotiation” and agents “alternately create, maintain, transform, and are constrained by social structures.” There is coordination between autonomously acting parts, and people coordinate themselves and each other in their social network rather than through “managers” who coordinate from the top or by a fixed and formalized set of rules.

In this study, Mintzberg's (1983) notion that standardization of skills is the dominant coordination mechanism for professional organizations such as hospitals is much less observed for organizing hospital-wide logistics. Interestingly, in his later work, Mintzberg (2012) advocates for more coordination through mutual adjustment and standardization of norms. Following Mintzberg's line of thinking, this raises the question of on what idea, belief or to what ends mutual adjustment is based. Perhaps there is expansive learning, a concept introduced by Engeström (2001) , in which engaged agents with differentiated tasks produce new patterns of activity, driven by their shared responsibility for patients. In addition, stabilizing the system appears to be an important motive for integration, especially because this is mainly done by agents with no formal responsibility for (large parts of) the system as a whole. This apparent need for stabilization may have to do with the absence of the concept of Takt time as a coordination mechanism. Takt time is the desired time between units of output, to be synchronized to the customers' demand, as described by Munavalli *et al.* (2017) . Takt time is considered important for the synchronization and integration of activities and for stabilizing the system ( Munavalli *et al.* , 2017 ). From this perspective, interactions in the social network could be there to respond to patient demand in the second instance, because the initial hospital-wide schedules are not based on patient demand. When surgeons plan patients, the open loop character of the system emerges and agents who observe this try to close these loops.

Perhaps this self-organizing, adaptive and learning organization is a good thing, as stated in literature and given the highly rated performance of Slingeland Hospital. There could, however, be a downside. The hospital's performance may be vulnerable and potentially unstable, leading to critical events, as mentioned by Ren *et al.* (2008) . The multitude of very detailed, conflicting or specific rules may hinder the overview of the system and hide possible open loops. In the SNA ( Van der Ham *et al.* , 2020 ), it was observed that the OTC capacity planner and the OTC day coordinator have a very central position in the network. This study shows that, together with the clinical bed planner, their integrative actions result from mutual adjustment and not from direct supervision. This can be a very challenging task for which extraordinary skills or even a certain personality is required. When these agents are absent or leave the hospital, the network may not only fall apart, but their knowledge of hospital-wide and local rules may disappear from the hospital, as most of those rules are unwritten. In addition, the lack of direct supervision by management may lead to a disconnection between the strategic and operational parts of the hospital. Relevant bottom-up feedback could be missed by top management, and operational agents may not respond to top-down management input, thus creating more open loops. This may hinder, for example, effective cost control of the hospital.

This exploratory case study provides an overview of the rules, coordination mechanisms and variables that are used for organizing the hospital-wide logistics for surgery patients. The main contribution of this study is that it reveals the rules and the coordination mechanisms that are used in a hospital, and it shows that the combination of these creates open loops, for which integration is achieved. In line with Yin (2014) , the findings should be used for “analytic generalization” ( Yin, 2014 , p. 40), and the lessons learned provide input to working hypotheses for future research. Clearly, the limitations of this study should be taken into account when drawing up hypotheses. The rules as described here may be temporary, and not universal, in time as well as given the environment of this particular hospital. Furthermore, from this study we cannot conclude how the coordination mechanisms relate to the hospital's performance.

Given the findings of this exploratory study, there are several issues we propose to explore further. First of all, we observe that the variability and uncertainty, which are inextricably characteristic of hospital logistics ( Aronsson *et al.* , 2011 ; Demeulemeester *et al.* , 2013 ; lega *et al.* , 2013 ; Hulshof *et al.* , 2012 ; Munavalli *et al.* , 2017 ; Van Merode *et al.* , 2004 ; Villa *et al.* , 2009 ; Zhu *et al.* , 2019 ), are managed through local rules and standards and by mutual adjustment processes. Integration is the result of actions of agents who try to stabilize the hospital system through mutual adjustment. We should further explore how integration and differentiation can be organized in hospitals in a more structural way. It is the question of what degree of integration and differentiation works and accordingly, how a closed loop system can be achieved within a certain network structure.

Further, we have to learn more about whether variability is the cause or the effect of the current way of working or both. We need to understand how and when to close the loops – in other words, how to use and facilitate mutual adjustment in these uncertain circumstances. In relation to this, it would be interesting to further explore which network structure and what rules and coordination mechanisms could facilitate Takt time management and thus facilitate stabilizing the hospital system. In addition, we need to explore how all this contributes to the hospital's performance.

We believe it is important to further develop Slingeland Hospital's logistical system in order to make it more robust, for example, by creating redundancy in the network, and possibly even improve its stability and performance. We should not leave the logistical organization of hospitals entirely up to the personal initiatives of some individuals nor discard informal mutual adjustment processes as a whole, for their apparent chaotic character. We propose three different approaches for future research.

First of all, if mutual adjustment is to be combined with standardization of norms ( Mintzberg, 2012 ), we need to understand what the norms of agents in hospitals are with regard to logistics. Mintzberg's norms ( 2012 ) refer to engagement in health care as a calling, but what this means for hospital logistics has to be specified further. A second approach is to study how the interplay between rules, coordination mechanisms, social network structures and hospital performance evolves over time. When circumstances change, both externally and internally, new rules may emerge and old ones be abolished, possibly as the result of a learning process. A third approach is to develop a multiagent system model in which the role of the OTC capacity planner is supported or even performed by an intelligent real-time scheduler, as proposed for outpatient clinics by Munavalli *et al.* (2020) . In this approach, the relation between coordination and performance is explored by developing formal negotiation processes and studying the effects of Takt time management in computer simulation models.

## Figures and Tables

**Table 1:** Rules and coordination mechanisms per task

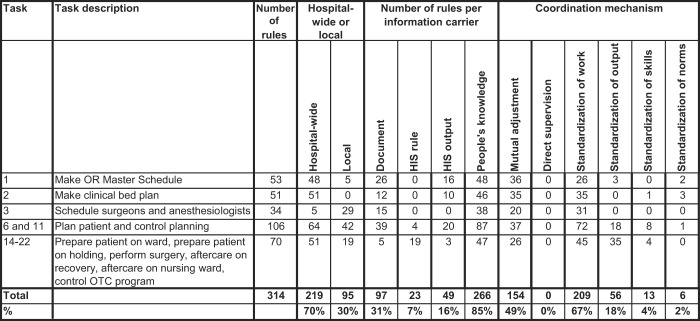

**Table 2:** Rules and coordination mechanisms over time

Time horizon	Tasks	% of rules		Coordination mechanism					
		Hospital wide	Local	Mutual adjustment	Direct supervision	Standardization of work	Standardization of output	Standardization of skills	Standardization of norms
3–6 months	1,2,3	75%	25%	66%	0%	67%	2%	1%	4%
1 day–12 weeks	6.11	60%	40%	35%	0%	68%	17%	8%	1%
Day of surgery	14,15,17,20,21,22	73%	27%	37%	0%	64%	50%	6%	0%

**Table 3:** Key output figures per medical discipline

Parameter #	Parameter	Dental surgery	Ear nose throat	Eye surgery	General surgery	Gynecology	Neurosurgery	Orthopedic surgery	Plastic surgery	Urology
*Sessions*										
1	Number of sessions per week (even/odd weeks)	2/4	4/5	4/4	21/23	4/3	2/2	14/13	4/3	6/6
2	Average percentage of sessions planned of OR master schedule	90%	79%	72%	93%	80%	100%	83%	89%	79%
3	Average session utilization	100%	94%	94%	88%	89%	85%	93%	83%	83%
4	Number of weeks deviated from OT master schedule	9	22	23	40	22	20	31	17	22
5	Number of weeks without sessions	2	2	6	0	1	18	0	1	1
6	Number of weeks with returned sessions	1	8	12	12	18	3	8	9	6
7	Number of weeks with extra sessions	4	2	0	27	6	5	2	3	7
*Surgeries*										
8	Number of surgeries performed in 2018	357	1069	941	3299	884	162	1742	515	876
9	Percentage planned surgeries	97%	97%	99.7%	68%	77%	99%	88%	86%	91%
10	Number of surgery types	21	22	11	130	30	10	69	31	44
11	Average number of surgeries per week	7	20	18	13	17	3	33	10	17
12	Variation number of surgeries per week (relative standard deviation)	43%	41%	49%	21%	35%	79%	30%	35%	38%
13	Average surgery time (min)	88	34	28	90	53	85	76	65	62
14	Variation surgery time (relative standard deviation)	26%	32%	29%	43%	32%	21%	28%	38%	29%

## Appendix Output figures

Appendix is available online for this article.

## References

[ref001] Aronsson , H. , Abrahamsson , M. and Spens , K. ( 2011 ), “ Developing lean and agile health care supply chains ”, Supply Chain Management: An International Journal , Vol. 16 , pp. 176 - 183 .

[ref002] Benchmark Coppa Consultancy ( 2012 ), “ Online commercial database ”, ( accessed 11 July 2018 ).

[ref003] Benham-Hutchins , M. and Clancy , T.R. ( 2010 ), “ Social networks as embedded Complex adaptive systems ”, The Journal of Nursing Administration , Vol. 40 , pp. 352 - 356 . 20798616 10.1097/NNA.0b013e3181ee42bc

[ref004] Beuving , J. and De Vries , G. ( 2015 ), Doing Qualitative Research: The Craft of Naturalistic Inquiry , Amsterdam University Press , Amsterdam .

[ref005] Borges , G.A. , Tortorella , G. , Rossini , M. and Portioli-Staudacher , A. ( 2019 ), “ Lean implementation in healthcare supply chain: a scoping review ”, Journal of Healthcare Organization and Management , Vol. 33 No. 3 , pp. 304 - 322 . 10.1108/JHOM-06-2018-0176 31122116

[ref006] Copp , M. ( 2005 ), “ Negotiated order ”, in Ritzer , G. (Ed.), Encyclopedia of Social Theory , Sage Publications .

[ref007] De Vries , J. and Huijsman , R. ( 2011 ), “ Supply Chain Management in health services: an overview ”, Supply Chain Management: An International Journal , Vol. 16 , pp. 159 - 165 .

[ref008] Demeulemeester , E. , Belien , J. , Cardoen , B. and Samundra , M. ( 2013 ), “ Operating room planning and scheduling ”, in Denton , B.T. (Ed.), Handbook of Operations Management: Methods and Applications. International Series in Operations Research and Management Science , Springer Science + Business Media , New York, NY .

[ref009] Drupsteen , J. , Van der Vaart , T. and Van Donk , D.P. ( 2013 ), “ Integrative practices in hospitals and their impact on patient flow ”, International Journal of Operations and Production Management , Vol. 33 , pp. 912 - 933 .

[ref010] Engeström , Y. ( 2001 ), “ Expansive Learning at Work: toward an activity theoretical reconceptualization ”, Journal of Education and Work , Vol. 14 , pp. 133 - 156 .

[ref011] Haythornthwaite , C. ( 1996 ), “ Social Network Analysis: an approach for the study of information exchange ”, LIST , Vol. 18 , pp. 323 - 342 .

[ref012] Hulshof , J.H. , Kortbeek , N. , Boucherie , R.J. , Hans , E.W. and Bakker , P.J.M. ( 2012 ), “ Taxonomic classification of planning decisions in healthcare: a structured review of the state of art in OR/MS ”, Health Systems , Vol. 1 , pp. 129 - 175 .

[ref013] Kilduff , M. and Tsai , W. ( 2003 ), Social Networks and Organizations , SAGE Publications , London , doi: 10.4135/9781849209915 .

[ref014] Kodner , D.L. and Spreeuwenberg , C. ( 2002 ), “ Integrated care: meaning, logic, applications and implications – a discussion paper ”, International Journal of Integrated Care , Vol. 2 No. 4 , doi: 10.5334/ijic.67 . PMC1480401 16896389

[ref015] Lawrence , P.R. and Lorsch , J.W. ( 1967 ), Organization and Environment: Managing Differentiation and Integration , Division of Research, Graduate School of Business Administration, Harvard University , Boston .

[ref016] Lega , F. , Marsilio , M. and Villa , S. ( 2013 ), “ An evaluation framework for measuring supply chain performance in the public healthcare sector: evidence from the Italian NHS ”, Production Planning and Control , Vol. 24 , pp. 931 - 947 .

[ref017] Litvak , N. , van Rijsbergen , M. , Boucherie , R.J. and van Houdenhoven , M. ( 2008 ), “ Managing the overflow of intensive care patients ”, European Journal of Operational Research , Vol. 185 No. 3 , pp. 998 - 1010 , doi: 10.1016/j.ejor.2006.08.021 .

[ref018] Ludwig , M. , Van Merode , G.G. and Groot , W. ( 2010 ), “ Principal agent relationships and the efficiency of hospitals ”, The European Journal of Health Economics , Vol. 11 , pp. 291 - 304 . 19655184 10.1007/s10198-009-0176-z PMC2860099

[ref019] Mintzberg , H. ( 1979 ), The Structuring of Organizations: A Synthesis of the Research , Prentice-Hall , Englewood Cliffs, New Jerssey, NJ .

[ref020] Mintzberg , H. ( 1983 ), Structure in Fives: Designing Effective Organizations , Prentice Hall , Englewoods Cliffs .

[ref021] Mintzberg , H. ( 2012 ), Managing the Myths of Health Care , Berrett-Koehler Publishers , Oakland . 23342753

[ref022] Monge , P.R. and Constractor , N. ( 2001 ), “ Emergence of communication networks ”, in Jablin , F.M. and Putman , L.L. (Eds), The New Handbook of Organizational Communication , Sage , Thousand Oaks , pp. 440 - 502 .

[ref023] Morgan , D. and Astolfi , R. ( 2015 ), “ Financial impact of the GFC: health care spending across the OECD ”, Health Economics, Policy and Law , Vol. 10 , pp. 7 - 19 . 25662194 10.1017/S1744133114000218

[ref024] Munavalli , J.R. , Rao , S.V. , Srinivasan , A. , Manjunath , U. and Van Merode , G.G. ( 2017 ), “ A robust predictive resource planning under demand uncertainty to improve waiting times in outpatient clinics ”, Journal of Health Management , Vol. 19 , pp. 563 - 583 . 30166799 10.1177/0972063417727627 PMC6097127

[ref025] Munavalli , J.R. , Rao , S.V. , Srinivan , A. and Van Merode , G.G. ( 2020 ), “ An Intelligent real-time scheduler for outpatient clinics: a multi-agent system model ”, Health Informatics Journal , Vol. 26 No. 4 , pp. 2383 - 2406 , doi: 10.1177/1460458220905380 . 32081068

[ref026] Parhankangas , A. , Ing , D. , Hawk , D.L. , Dane , G. and Kosits , M. ( 2005 ), “ Negotiated order and network form organizations ”, Systems Research and Behavioral Science , Vol. 22 , pp. 431 - 452 .

[ref027] Provan , K.G. and Sebastian , J.G. ( 1998 ), “ Networks within networks: service link overlap, organizational cliques, and network effectiveness ”, The Academy of Management Journal , Vol. 41 No. 4 , pp. 453 - 463 .

[ref037] Przywara , B. ( 2010 ), Projecting Future Health Care Expenditure at European Level: Drivers, Methodology and Main Results. European Economy - Economic Papers 2008 – 2015 , Directorate General Economic and Financial Affairs (DG ECFIN), European Commission .

[ref028] Ren , Y. , Kiesler , S. and Fussell , S.R. ( 2008 ), “ Multiple group coordination in complex and dynamic task environments: interruptions, coping mechanisms, and technology recommendations ”, Journal of Management Information Systems , Vol. 25 , pp. 105 - 130 .

[ref029] Uddin , M.S. and Hossain , L. ( 2011 ), “ Social networks enabled coordination model for cost management of patient hospital admissions ”, Journal for Healthcare Quality , Vol. 33 , pp. 37 - 48 . 10.1111/j.1945-1474.2011.00118.x 23845132

[ref030] Van der Ham , A. , Boersma , H. , Van Raak , A. , Ruwaard , D. and Van Merode , G.G. ( 2018 ), “ Identifying Logistical Parameters in Hospitals: does literature reflect integration in hospitals? A scoping study ”, Health Services Management Research , Vol. 32 No. 3 , pp. 158 - 165 , doi: 10.1177/0951484818813488 . 30463453 PMC7324119

[ref031] Van der Ham , A. , Van Merode , F. , Ruwaard , D. and Van Raak , A. ( 2020 ), “ Identifying Integration and Differentiation in a hospital's logistical system: a social network analysis of a case study ”, BMC Health Services Research , Vol. 20 , p. 857 , doi: 10.1186/s12913-020-05514-w . 32917198 PMC7488445

[ref032] Van Hulst , B. and Blank , J. ( 2017 ), “ Nederlandse ziekenhuizen te groot voor verdere schaalvoordelen ”, Economisch-Statistische Berichten , Vol. 102 No. 4749 , pp. 226 - 228 .

[ref033] Van Merode , G.G. , Groothuis , S. and Hasman , A. ( 2004 ), “ Enterprise resource planning for hospitals ”, International Journal of Medical Informatics , Vol. 73 , pp. 493 - 501 . 15171978 10.1016/j.ijmedinf.2004.02.007

[ref034] Villa , S. , Barbieri , M. and Lega , F. ( 2009 ), “ Restructuring patient flow logistics around patient care needs: implications and practicalities from three critical cases ”, Health Care Management Science , Vol. 12 No. 2 , pp. 155 - 165 . 19469455 10.1007/s10729-008-9091-6

[ref035] Yin , R.K. ( 2014 ), Case Study Research – Design and Methods , 5th ed. , Sage Publications , Thousand Oaks .

[ref036] Zhu , S. , Fan , W. , Yang , S. , Pei , J. and Pardalos , P.M. ( 2019 ), “ Operating room planning and surgical case scheduling: a review of literature ”, Journal of Combinatorial Optimization , Vol. 37 , pp. 757 - 805 .

