# Supplementary material for: Explaining integration and differentiation by identifying the rules and coordination mechanisms in a hospital's logistical system
Source: J Health Organ Manag. 2021 Feb 23;35(9):66–84. doi: 10.1108/JHOM-06-2020-0236 (PMC9251638; doi:10.1108/JHOM-06-2020-0236)
Supplement: Supplementary file 1 [file jhealthorganmanag-35-0066.docx]

**Appendix A: Output figures**

**Table A1:** Number of surgery patients per location

**Table A2:** Key output figures per medical discipline

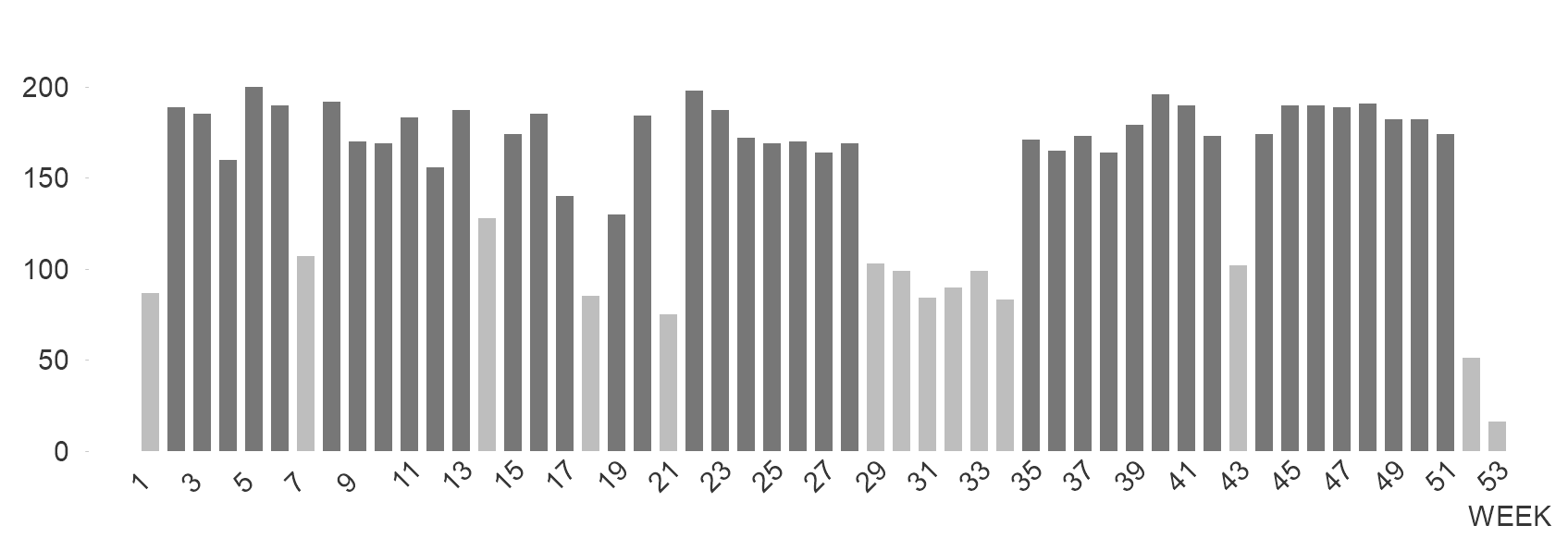


**Figure A1:** Number of surgeries per week


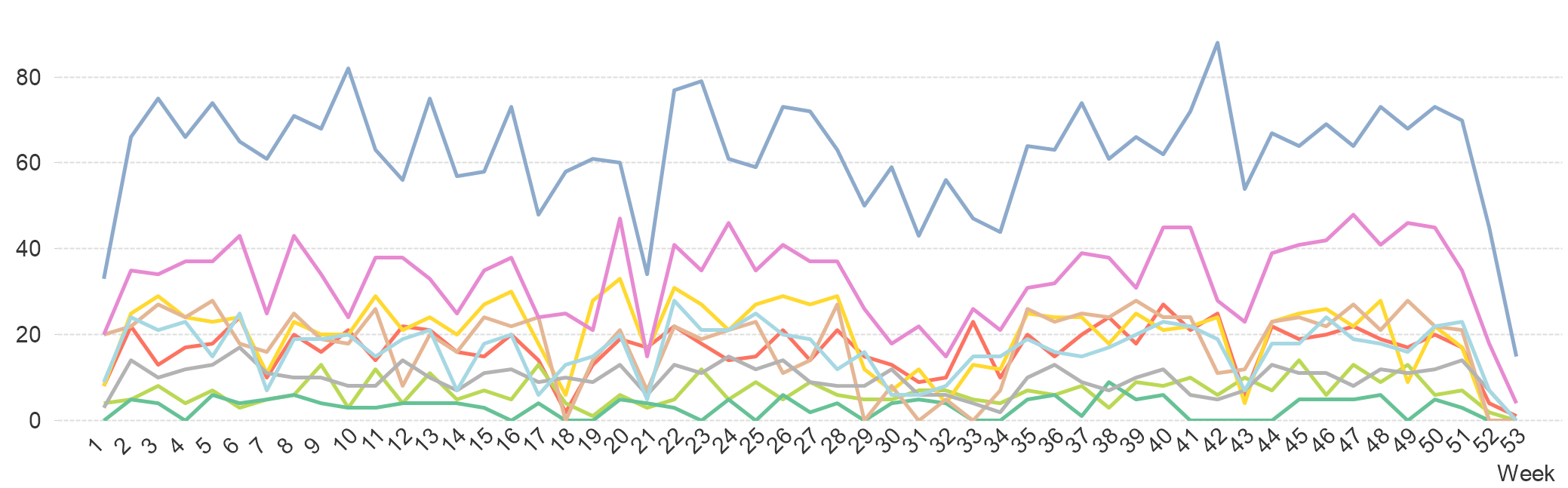


**Figure A2:** Number of surgeries per week per medical discipline

**Appendix B: The tasks, agents, interactions and coordination mechanisms in the social network**

***Task 1: Make OR Master Schedule***

| **Task description** | The Operating Room (OR) master schedule is made in the Tactical Planning Meeting (TPM). Three cluster managers, who are responsible, respectively, for outpatient, inpatient departments and the Operating Theatre Complex (OTC), nine surgeons, one anesthesiologist and the OTC capacity planner participate in this meeting. The OR master schedule is prepared by four participants of the TPM, who then propose the scheme to the entire TPM. The OR master schedule is then presented to the OTC commission. The OTC commission discusses and advises OTC management on planning, staff and budget issues. The OTC commission includes one surgeon from every surgical discipline, the cluster manager responsible for the OTC and the OTC capacity planner. When the OR master schedule is final, the OTC capacity planner informs all outpatient secretaries on the changes made. |
| --- | --- |
| **Time horizon** | 12 to 20 weeks ahead |
| **Frequency** | 4 times a year |

**Interactions**

**Rules for task performance**

***Task 2: Make Clinical Bed Plan***

| **Task description** | In the clinical bed plan, beds are assigned to a medical discipline for each nursing department. The clinical bed plan is established in consultation between the OTC capacity planner, one secretary from the nursing department and the two team leaders of the three nursing wards who host most surgery patients. The team leaders of these nursing wards are also involved in making the clinical bed plan, but one nurse ward secretary has the informal role of ‘clinical bed plan boss’. |
| --- | --- |
| **Time horizon** | 12 to 20 weeks ahead |
| **Frequency** | 4 times a year |

**Interactions**

**Rules for task performance**

***Task 3: Schedule surgeons and anesthesiologists***

| **Task description** | The surgeons of each outpatient department and anesthesiologists make schedules for themselves and allocate surgeons to the time slots in the OR master schedule. The scheduler of each medical discipline group proposes the schedule and discusses it with the surgeons. In some outpatient departments the secretary of the outpatient department is involved in this. |
| --- | --- |
| **Time horizon** | 10 weeks ahead |
| **Frequency** | Variable |

 **Interactions**

**Rules for task performance**

***Tasks 6 and 11: Plan patient and control planning***

| **Task description** | 6) | The planning for surgeries takes place at one of the nine outpatient clinics. The secretary puts the patient on the waiting list or sets a surgery date right away. There can be interaction between secretaries and the OTC capacity planner on specific surgery requirements or when the OR master schedule is almost filled. Surgeons also email or phone the OTC capacity planner for specific patient cases that require tuning. |
| --- | --- | --- |
|  | 11) | The planning is checked and revised in the process leading to surgery. The preoperative secretaries interact with all outpatient secretaries on whether everything is arranged for the surgery. In the weekly ‘Tuesday morning’ meeting, the planning for the upcoming week is discussed among the outpatient secretaries, the ward team leaders and the OTC capacity planner. Also, a weekly bed meeting takes place between ward team leaders, a nurse and the OTC capacity planner. All checks for the next day’s OR program are made in this meeting. The OTC capacity planner views the daily OTC schedule the day before to determine the exact sequence of the operations. When everything is checked, she informs the OTC day coordinator regarding any specific details in the next day’s OTC program. |
| **Time horizon** | 6)  11) | 6 days to 20 weeks ahead  1 day to 2 weeks ahead |
| **Frequency** | 6)  11) | Daily  Daily |

 **Interactions**

**Rules for task performance**

***Tasks 14,15,17,20,21 and 22: Prepare patient on ward, holding, perform surgery and aftercare on recovery and nursing ward, control OTC program***

| **Task description** | 14) | The patient is admitted to one of the nursing wards. The nurse anesthetist then calls the nurse to indicate that premedication should be given to the patient, mostly two hours before the expected starting time of the surgery. The nurse anesthetist makes a second call to the nurse to say that the patient is to be taken to holding area. |
| --- | --- | --- |
|  | 15) | The ward nurse hands the patient over to one of the holding nurses. The holding nurse prepares the patient for surgery. When it is time to go to the OR, the nurse anesthetist enters the holding area and the holding nurse hands the patient over to the nurse anesthetist. |
|  | 17) | The nurse anesthetist mentions any relevant details about the patient to the rest of the OR team. The anesthesiologist administers anesthetics prior to the surgery and leaves once the patient is asleep. The surgery is performed by the surgeon, assisted by the OR nurses. The nurse anesthetist monitors the patient and calls the anesthesiologist if necessary. |
|  | 20) | After surgery the patient is transferred to recovery by the nurse anesthetist. The transfer is performed between the nurse anesthetist and the recovery nurse. The recovery nurses interact with the anesthesiologists regarding the medication policy if necessary. |
|  | 22) | There are several tasks, performed at various moments in time, making sure that the surgeries that are scheduled for one day are performed well and on time. There is daily start of day meeting between the OTC day coordinator and the three OTC team leaders, in which expectations and special surgeries are discussed. During the course of the day the OTC day coordinator monitors the progress of each OR. The OTC day coordinator communicates with the anesthesiologist or surgeons about any alterations required in the OR scheme. |
|  |  |  |
| **Time horizon** | All tasks) | Day of surgery |
| **Frequency** | All tasks) | Daily |

**Interactions**

**Rules for task performance**
